# The Midland and North of England Stillbirth Study (MiNESS)

**DOI:** 10.1186/1471-2393-14-171

**Published:** 2014-05-21

**Authors:** Jayne Platts, Edwin A Mitchell, Tomasina Stacey, Bill L Martin, Devender Roberts, Lesley McCowan, Alexander E P Heazell

**Affiliations:** 1 Maternal and Fetal Health Research Centre, Research Floor (5th floor), Institute of Human Development, University of Manchester, Maternal and Fetal Health Research Centre, St Mary’s Hospital, Oxford Road, Manchester M13 9WL, UK; 2 Department of Obstetrics, St Mary’s Hospital, Oxford Road, Manchester M13 9WL, UK; 3 Department of Paediatrics, University of Auckland, Private Bag 92019, Auckland 1142, New Zealand; 4 Mid Yorkshire Hospitals NHS Trust, Dewsbury & District Hospital, Halifax Road, Dewsbury WF13 4HS, UK; 5 Birmingham Women’s Hospital NHS Foundation Trust, Mindelsohn Way, Edgbaston, Birmingham B15 2TG, UK; 6 Liverpool Women’s Hospital NHS Foundation Trust, Crown Street, Liverpool L8 7SS, UK; 7 Department of Obstetrics & Gynaecology, University of Auckland, Private Bag 92019, Auckland 1142, New Zealand

**Keywords:** Stillbirth, Perinatal mortality, Perinatal death, Risk factors, Sleep position, Reduced fetal movements, Fetal growth restriction

## Abstract

**Background:**

The United Kingdom has one of the highest rates of stillbirth in Europe, resulting in approximately 4,000 stillbirths every year. Potentially modifiable risk factors for late stillbirths are maternal age, obesity and smoking, but the population attributable risk associated with these risk factors is small.

Recently the Auckland Stillbirth Study reported that maternal sleep position was associated with late stillbirth. Women who did not sleep on their left side on the night before the death of the baby had double the risk compared with sleeping on other positions. The population attributable risk was 37%. This novel observation needs to be replicated or refuted.

**Methods/Design:**

Case control study of late singleton stillbirths without congenital abnormality. Controls are women with an ongoing singleton pregnancy, who are randomly selected from participating maternity units booking list of pregnant women, they are allocated a gestation for interview based on the distribution of gestations of stillbirths from the previous 4 years for the unit. The number of controls selected is proportional to the number of stillbirths that occurred at the hospital over the previous 4 years.

Data collection: Interviewer administered questionnaire and data extracted from medical records. Sample size: 415 cases and 830 controls. This takes into account a 30% non-participation rate, and will detect an OR of 1.5 with a significance level of 0.05 and power of 80% for variables with a prevalence of 57%, such as non-left sleeping position.

Statistical analysis: Mantel-Haenszel odds ratios and unconditional logistic regression to adjust for potential confounders.

**Discussion:**

The hypotheses to be tested here are important, biologically plausible and amenable to a public health intervention. Although this case–control study cannot prove causation, there is a striking parallel with research relating to sudden infant death syndrome, where case–control studies identified prone sleeping position as a major modifiable risk factor. Subsequently mothers were advised to sleep babies prone (“Back to Sleep” campaign), which resulted in a dramatic drop in SIDS. This study will provide robust evidence to help determine whether such a public health intervention should be considered.

**Trial registration number:**

NCT02025530

## Background

The death of an unborn child is a tragic public health problem which currently affects approximately 2.6 million families annually worldwide [1]. Unlike other avoidable causes of maternal and child death the rate of decline of late stillbirth (at or over 28 weeks gestation) in high income countries (HICs) has slowed in recent decades [1].

The United Kingdom has one of the highest rates of stillbirth in Europe, ranking 33^rd^ out of 35 high-income countries [1]. The last report from the Centre for Maternal and Child Enquiries (CMACE) highlights a slow decline in the stillbirth rate in the UK from 5.4 per 1000 total births in 2000 to 5.2 per 1000 total births in 2009, [2]. This decline in stillbirth rate is supported by Gardosi [3] who attributes the reduction to the uptake of accredited training in fetal growth assessment. The Lancet Stillbirth Series [1,3,4] has highlighted the silent but prevalent public health problem of stillbirth and together with the UK Stillbirth and Neonatal Death Charity (Sands) and the Royal College of Obstetricians and Gynaecologists (RCOG) has called for research to address these unacceptably high rates.

### Established risk factors for late stillbirth

Current established risk factors for late stillbirth in high income countries are well documented and have largely been identified from population-level epidemiological studies [5]. These include: advanced maternal age (>35 years) [6], obesity [7], smoking [8], reduced antenatal care attendance [9], low socio-economic status [9], women from black and minority ethnic groups [2], reduced fetal movements (RFM) [10] and small for gestational age (SGA) infants [11]. A meta-analysis of population-based studies found the three most important modifiable risk factors for stillbirth were: obesity (population attributable risk (PAR) 8-18%), advanced maternal age (population attributable risk 6-8%) and smoking (population attributable risk 4-7%) [5]. Of these, only cigarette smoking can realistically be addressed by women during pregnancy. Currently in the UK, there are efforts to address increased identification [3] and subsequent management of SGA infants and mothers following presentation with RFM through the implementation of guidelines [12,13]. To date there has been limited research investigating novel, modifiable factors which have the potential to advance knowledge and address important gaps in the field of stillbirth research.

### Novel modifiable factors and late stillbirth

Although adults spend about a third of their lives asleep there had not been any studies that examined a potential relationship between maternal sleep practices and risk of late stillbirth prior to The Auckland Stillbirth Study [14]. This reported that women who did not go to sleep on their left side on the night before experiencing a late stillbirth, had a two-fold increase in risk compared with those who did go to sleep on their left [14]. This effect persisted after adjustment for confounders such as obesity. The PAR for non-left sleep position in this study was 37%, greater than the PARs of obesity, advanced maternal age and smoking combined [4]. In addition, women who got up to the toilet once or less on the last night before the stillbirth, compared to those who got up more often, were also at higher risk of late stillbirth, as were those who regularly slept during the day in the last month, compared to those who did not.

The Auckland Stillbirth Study also asked women about fetal activity preceding stillbirth. In common with previous studies [15,16], a two-fold increase in late stillbirth was reported in women perceiving reduced fetal movements (RFM) [17]. Although, maternal perception of reduced fetal activity is often used to highlight pregnancies that require further investigation [18], the RCOG guideline on the management of RFM highlighted the need for further studies to understand how maternal perception of reduced fetal activity can be used in stillbirth prevention [12]. Another novel finding of Stacey et al. was the association between a single episode of vigorous fetal activity and late stillbirth [17].

In support of the findings of Stacey et al. The Sydney Stillbirth Study recruited 295 women from eight hospitals around Australia; found that women who slept on their backs were four times more likely to have a stillbirth [19]. A small survey of maternal sleep practices in Ghana also identified supine sleeping position with an increased risk of stillbirth (stillbirth rate (3/19) 15.8% in those sleeping supine and (6/197) 3.0% in those in non-supine position; odds ratio (OR) 8.0; 95% CI 1.5-43.2) [20]. However, both these studies were underpowered to identify right sided sleep position as a risk factor and were unable to examine whether or not sleep position was a particular risk-factor for already compromised babies (FGR and RFM). The proposed study will address these issues in an ethnically and socially diverse population.

### Unanswered questions from the Auckland Stillbirth Study

Publication of the Auckland Stillbirth Study and related correspondence in 2011 was accompanied by an editorial by Chappell and Smith which raised several questions [21]; the first concerned reporting bias; specifically those higher educated women would have greater access and uptake of knowledge about sleep position. This seems unlikely as this was the first published report of the association between stillbirth and sleep position. They also raised the issue of reverse causality. The association between longer sleep and not getting up at night might reflect RFM, an indication of a compromised baby, and this compromise reflects the cause of stillbirth, not duration of sleep duration or not rising at night [21]. In subsequent correspondence Frøen et al. also raised the possibility that the study findings are due to confounding by FGR or RFM [22]. They argued that in cases of FGR the smaller uterus might result in a reduction in the normal preference for a lateral sleeping position in late pregnancy, less bladder compression (not having to get up to the toilet as frequently), and thus better and longer sleep duration. In their reply, the Auckland Stillbirth Study team undertook additional analyses adjusting for these factors and the associations between maternal sleep practices and stillbirth did not change.

There was general agreement that before intervention studies can be planned further observational studies are necessary to confirm or refute the findings of the Auckland Stillbirth Study and investigate potential underlying mechanisms. Here, we propose a study which will achieve this in an ethnically and socially diverse population where the effects of FGR and RFM can also be studied.

### Hypotheses

During this study we will test the following hypotheses:

1. Maternal left sided sleep position reduces the risk of late stillbirth.

2. Supine sleep position increases the risk of late stillbirth

3. Increased maternal sleep duration and sleeping during the day increase the risk of late stillbirth.

4. Maternal perception of RFM, prior to fetal death, increases the risk of late stillbirth.

5. Non-left sleep position, in conjunction with prolonged sleep, increases the risk of late stillbirth substantially (that is, there is an interaction between sleep position and prolonged sleep).

6. Non-left sleep position, in conjunction with a compromised baby (for example, FGR, smoke or drug exposure, RFM) increases the risk of late stillbirth.

### Methods/Design

Modifiable risk factors associated with late stillbirth.

### Study design

After due consideration a case control study has been selected as the most appropriate and efficient design, this methodology has been used to study relatively rare disorders such as stillbirth and addresses the aims of this study.

Alternative study designs considered by the study team included a cohort study of pregnant women. However, the size of study required is unfeasibly large; assuming a late stillbirth rate of 3/1,000 births, to identify 291 cases, 97,000 women would need to be recruited at 28 weeks gestation. For a randomised controlled trial (RCT) the requisite sample size would also be prohibitively large. Assuming the absolute stillbirth rate for the non-left sided position is 3.9/1,000 births and the risk is halved by left sided sleeping and that all participants are able to change their sleep position 118,000 pregnant women would be required in each group (power = 80%, p = 0.05). Asking pregnant women who normally sleep on their left to sleep in another position would be unethical as we believe this would increase their risk of stillbirth, thus only women who normally slept on the right or back could be randomised, increasing the sample size by over two fold. Therefore, it is unlikely an adequately powered RCT of individual women will ever be conducted. Importantly, this was also the case for the “back to sleep” campaign for sudden infant death syndrome which was never evaluated by RCT.

### Study setting

Ethical approval has been successfully obtained from Central Manchester Research Ethics Committee (13/NW/0874). Participants will be recruited from a total of 35 maternity units around the Midlands and North of England. The units have been chosen for location irrespective of size or any local demographics. Recruitment to the project and administration of the questionnaire will be carried out locally by research midwives from participating units. These will be supported by three research midwife co-ordinators based in Manchester, Yorkshire and Birmingham.

### Time scale

September 2013 to March 2014: Study set up, obtain ethical and research approval.

April 2014 to December 2015: Recruitment and data collection from all sites.

January 2016 to August 2016: Data analysis, report writing, dissemination of research findings.

### Sample size

If the prevalence of a risk factor in the controls is between 30% and 60% (the prevalence of non-left sleep position in the Auckland Stillbirth Study was 57%) we would need a sample of 291 cases and 582 controls to detect an odds ratio of 1.5 with a significance level of 0.05 and power of 80%. This sample size would also allow us to detect an interaction with an OR of 2.5. The number of women approached to participate in the study needs to be greater than this as we anticipate a proportion of women will not participate in the study, in the Auckland Stillbirth Study this was 28%. Assuming 30% non-participation rate we need to approach 415 eligible cases and 830 eligible controls.

From the data collected during the recruitment of participating units there are approximately 170 normally formed singleton stillbirths in the Greater Manchester region per year, 90 in the Mersey region, 190 in Yorkshire and 160 in the West Midlands this would give an potential annual recruitment of 610 cases. Due to the unpredictability of stillbirth occurrence we have allowed 18–24 months for recruitment.

### Inclusion criteria

#### Cases

Women with singleton pregnancies ending in late stillbirth (with no congenital abnormality) will be recruited from participating maternity units (n ~ 35) in the Midlands and North of England led by four centre’s in Liverpool, Manchester, Yorkshire and Birmingham.

#### Controls

The control group are women with an ongoing pregnancy at a group matched gestation. Women who subsequently deliver an infant with a congenital abnormality will be excluded from the study analysis. This method for control selection overcomes the limitations of previous studies which used gestation matched live birth cohorts which includes many infants who have risk factors for preterm birth.

### Exclusion criteria

Women with the following criteria will not be eligible for participation in the study:

● Fetus known to have a significant congenital anomaly (as defined by the NHS fetal anomaly screening programme (FASP) – Anencephaly, Open spina bifida, Cleft lip, Diaphragmatic hernia, Gastroschisis, Exomphalos, Serious cardiac abnormalities, Bilateral renal agenesis, Lethal skeletal dysplasia, Edward’s syndrome (trisomy 18), Patau’s syndrome (trisomy 13). http://fetalanomaly.screening.nhs.uk/fetalanomalyresource/whats-in-the-hexagons1/about-the-scan/about-the-11-key-conditions)

● Multiple pregnancy

● Maternal age <16 years

● Women unable to give informed consent.

### Participant recruitment

Prior to their discharge from their maternity unit, eligible women who have had a stillbirth (cases) will be given a brief written description of the study by their midwife or doctor and asked whether a research midwife could contact them to discuss the study. If they agree, a research midwife will contact the woman and explain the study in more detail. If the woman consents to participate, a time and place for the interview will be arranged. Interviews will be conducted by trained research midwives. The co-ordinating midwives based in Manchester, Yorkshire and Birmingham will be responsible for training local research midwives in the structured interview format and in providing an appropriate interview environment. This link will also facilitate ongoing support for local research midwives. A similar recruitment system was established through the Auckland Stillbirth Study where formal feedback from participants was positive and no negative feedback was received regarding recruitment [23]. The investigators already have experience of recruiting parents for studies after stillbirth, which will ensure potential distress is minimised. Rather than being reluctant to participate, bereaved parents are keen to participate in research, even that closely related to their time of loss [24]. We will examine the views of women regarding their participation in this research project in a small nested cohort qualitative study.

**Figure 1:**
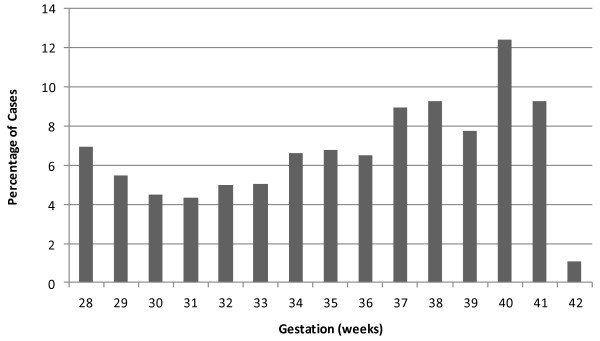
**Distribution of gestational age of late singleton stillbirths (excluding those with congenital abnormalities) in the study units (2009–2012).** Controls are group matched to these gestations.

Controls will be recruited using a group matching technique, based on the previous four years birth and stillbirth figures, they will be randomly selected from the participating hospitals booking list of pregnant women (Figure 1). The control participants will be approached by their community midwife or research midwife, who will give a brief explanation of the study. If women agree to participate, a time and place for the interview will be arranged. As for the cases we will examine the views of women in the control group regarding their participation in this research project in a small qualitative nested cohort study – this will particularly focus on the impact of recruiting women with an apparently healthy pregnancy to a study about stillbirth.

### Data collection

Data collection is by interviewer-administered questionnaire which has been developed from the questionnaire used in the Auckland Stillbirth Study. Participants will be interviewed face to face by research midwives with interpreters if required. For cases the interview will occur as close to the time of stillbirth as possible, usually within 1–3 weeks. We hope to reduce the interval between stillbirth and the interview from that in the Auckland Stillbirth Study. The research midwife will refer to the study’s distress policy if women become upset or distressed during the interviews. Experience from Auckland and Sydney found that the questions did not increase maternal anxiety, indeed many stillbirth mothers welcomed the opportunity to talk about their experience [17].

In addition to the questionnaire, data will also be extracted from medical records. The dataset will include: demographic and socio-economic factors; maternal general health; medications; smoking; drug use (pharmaceutical and recreational); diet; body mass index (BMI) and pregnancy weight gain; fetal movements; stress assessment; sleep disordered breathing; sleep positions and other sleep habits.

### Analysis

Analysis will be carried out using the standard Mantel-Haenszel odds ratio analysis used in case–control studies. Conditional logistic regression will be used to adjust for potential confounders (including gestation and obstetric hospital of birth) and to determine the presence of interactions, particularly to address the interaction between FGR, RFM and sleep position.

## Discussion

Stillbirth is a tragic and prevalent outcome of pregnancy associated with both psychological and physical morbidity and social cost to the affected families and broader community. Stillbirth may be associated with a variety of underlying conditions. However, the terminal event is frequently not appreciated and stillbirth is likely to result from multifactorial processes [25].

The hypotheses to be tested here are important, biologically plausible and amenable to a public health intervention. In some cases, RFM is associated with evidence of placental dysfunction, which is also seen in stillbirth [26,27]. Non-left sided and especially supine sleep position could be associated with aorto-caval compression reducing maternal cardiac output and uterine perfusion [28]. Thus, this represents a pivotal opportunity with respect to stillbirth research. This project has the potential to:

● advance knowledge about novel, modifiable risk factors for late stillbirth, and

● translate into substantial improvements in rates of late stillbirth in the UK and internationally should the hypothesis be proved.

Current understanding of the causes and prevention of late stillbirth is similar to the state of knowledge regarding sudden infant death syndrome (SIDS) two decades ago. In the late 1980s several studies demonstrated an increased risk of SIDS when infants slept prone [29-31]. Subsequently, mothers were advised to place their babies “Back to Sleep” which resulted in a dramatic reduction in SIDS [32-35]. Similar principles may be applicable to late stillbirth; certainly any effect in stillbirth is likely to require evaluation in similar population-based intervention studies to see an effect on perinatal mortality.

It is imperative that prior to distribution of information public health interventions to reduce stillbirths are based upon robust evidence. The MiNESS will provide robust evidence which will help to determine whether such an intervention study should be considered.

## Abbreviations

BMI: Body mass index; CMACE: The Centre for Maternal and Child Enquiries; CI: Confidence interval; FGR: Fetal growth restriction; OR: Odds ratio; PAR: Population attributable risk; RCOG: Royal College of Obstetricians and Gynaecologists; RCT: Randomised controlled trial; RFM: Reduced fetal movements; Sands: The UK Stillbirth and Neonatal Death Charity; SIDS: Sudden Infant Death Syndrome; SGA: Small for gestational age.

## Competing interests

None of the authors have any financial or non-financial competing interests to disclose.

## Authors’ contributions

AH, TS, BM, DR, EM & LM contributed to all aspects of the study design. AH has overall responsibility for the study. TS, EM & LM provided study details of TASS. JP & AH drafted the protocol and obtained ethical approvals. EM has developed the recruitment strategy and data analysis plan. JP conceptualized and developed the staff training programmes used. JP & AH are responsible for the drafting of the manuscript. All authors gave approval for the final version of the manuscript.

## Pre-publication history

The pre-publication history for this paper can be accessed here:

http://www.biomedcentral.com/1471-2393/14/171/prepub
